# Preparation, characterization and photocatalytic behavior of WO_3_-fullerene/TiO_2 _catalysts under visible light

**DOI:** 10.1186/1556-276X-6-459

**Published:** 2011-07-20

**Authors:** Ze-Da Meng, Lei Zhu, Jong-Geun Choi, Chong-Yeon Park, Won-Chun Oh

**Affiliations:** 1 Department of Advanced Materials Science & Engineering, Hanseo University, Seosan, Chungnam, 356-706, South Korea

## Abstract

WO_3_-treated fullerene/TiO_2 _composites (WO_3_-fullerene/TiO_2_) were prepared using a sol-gel method. The composite obtained was characterized by BET surface area measurements, X-ray diffraction, scanning electron microscopy, energy dispersive X-ray analysis, transmission electron microscopy, and UV-vis analysis. A methyl orange (MO) solution under visible light irradiation was used to determine the photocatalytic activity. Excellent photocatalytic degradation of a MO solution was observed using the WO_3_-fullerene, fullerene-TiO_2_, and WO_3_-fullerene/TiO_2 _composites under visible light. An increase in photocatalytic activity was observed, and WO_3_-fullerene/TiO_2 _has the best photocatalytic activity; it may attribute to the increase of the photo-absorption effect by the fullerene and the cooperative effect of the WO_3_.

## Introduction

Textile manufacturing involves several processes which generate large quantities of wastewaters. These effluents are highly variable in composition with relatively low biochemical oxygen demand and high chemical oxygen demand contents and are typically characterized as follow: first: strong color due to residual dyes, second: recalcitrance due to the presence of compounds such as dyes, surfactants, and sizing agents; and third: high salinity, high temperature, and variable pH [1-3]. The textile effluents effective treatment usually requires a combination of various physical, chemical, and biological technologies. Some studies researched the treatment of model solutions containing various commercial dyes with emphasis on azo dyes since these are extensively used in dyeing processes. These azo dye molecules are chemically stable and hardly biodegradable aerobically. Most attention has been paid on the oxidative degradation of MB and MO representative mono-azo dyes by oxidation processes [4,5]. TiO_2 _is the most widely used photocatalyst far effective decomposition of organic compounds in air and water under irradiation of UV light with wavelength shorter than corresponding to its band gap energy, due to its relatively high photocatalytic activity, biological and chemical stability, low cost, non-toxic nature, and long-term stability. However, the photocatalytic activity of TiO_2 _(the band gap of anatase TiO_2 _is 3.2 eV and it can be excited by photons with wavelengths below 387 nm) is limited to irradiation wavelengths in the UV region [6,7]. However, only about 3% to 5% of the solar spectrum falls in this UV range. This limits the efficient utilization of solar energy for TiO_2_. Some problems still remain to be solved in its application, such as the fast recombination of photogenerated electron-hole pairs. Therefore, improving photocatalytic activity by modification has become a hot topic among researchers in recent years [8,9].

For the improvement of the photocatalytic activity of TiO_2_, TiO_2 _has been coupled with other semiconductors such as SnO_2 _[10] which can induce effective charge separation by trapping photogenerated electrons. TiO_2 _coupled with other semiconductors has been reported to perform both the abovementioned functions. This has been realized by coupling the WO_3 _[11] semiconductor with TiO_2_. Because of its band gap (*E*_g _= 2.6 eV to approximately 3.0 eV) [12], WO_3 _mainly absorbs in the near ultraviolet and blue regions of the solar spectrum. As a basic function, WO_3 _has a suitable conduction band potential to allow the transfer of photogenerated electrons from TiO_2 _facilitating effective charge separation. However, in practical applications, the photoelectrical properties and photocatalytic efficiency of WO_3 _require improvement.

C_60 _has attracted considerable interest for its interesting properties owing to the delocalized conjugated structures and electron-accepting ability. One of the most remarkable properties of C_60 _in electron-transfer processes is that it can efficiently arouse rapid photoinduced charge separation and relatively slow charge recombination [13]. Therefore, a combination of photocatalysts and C_60 _might provide an ideal system to achieve enhanced charge separation by photoinduced electron transfer. Some fullerene-donor linked molecules on an electrode were reported to exhibit excellent photovoltaic effects upon photo-irradiation.

A conjugated two-dimensional π-system is suitable not only for synthetic light-harvesting systems but also for efficient electron transfer because the uptake or release of electrons results in minimal structural and solvation change upon electron transfer. Fullerenes contain an extensively conjugated three-dimensional π-system and are described as having a closed-shell configuration consisting of 30 bonding molecular orbitals with 60 π-electrons. This material is also suitable for efficient electron-transfer reduction because of the minimal changes in structure and salvation associated with electron transfer [14,15].

Unfortunately, deposited metal particles or coupled with other semiconductors only serve as electron trapping agent, or transfer of photogenerated electrons and are not effective to enhance the adsorption of the pollutants. Fullerene-treated TiO_2 _coupled with other semiconductors has been reported to perform both the abovementioned functions [16]. In addition, C_60 _is one of the promising materials because of its band gap energy, about 1.6 to 1.9 eV. It has strong absorption in the ultraviolet region and weak but significant bands in the visible region. In general, the coupled systems exhibit higher degradation rate as well as the increased extent of degradation [17]. The studies for comparing the coupled semiconductors with visible light, however, are scarce.

In this paper, WO_3_-treated fullerene, fullerene-supported TiO_2_, and WO_3_-fullerene/TiO_2 _were synthesized and exhibited enhanced vis-photocatalytic activities compared to the pure TiO_2_. This study focused on the fabrication and characterization of WO_3_-fullerene/TiO_2 _composite in a preparation procedure. Structure variations, surface state, and elemental compositions were examined for the preparation of WO_3_-fullerene/TiO_2 _composites. X-ray diffraction (XRD), scanning electron microscopy (SEM), energy dispersive X-ray (EDX), transmission electron microscopy (TEM), and UV-visible (UV-vis) were used to characterize these new photocatalysts. The catalytic efficiency of the WO_3_-fullerene/TiO_2 _composite was evaluated by the photo degradation of methyl orange (MO, C_14_H_14_N_3_NaO_3_S).

## Materials

Benzene (99.5%) and ethyl alcohol were purchased as reagent-grade from Duksan Pure Chemical Co. (Ansan-si, Gyeonggi-do, South Korea) and Daejung Chemical Co. (Gwangju-si, Gyeonggi-do, South Korea) and were used as received. Crystalline fullerene [C_60_] powder (99.9% purity from Tokyo Kasei Kogyo Co. Ltd., Tokyo, Japan) was used as the carbon matrix. Titanium(IV) *n*-butoxide (TNB, C_16_H_36_O_4_Ti) as the titanium source for the preparation of the WO_3_-fullerene/TiO_2 _composites was purchased as reagent-grade from Acros Organics (Morris Plains, NJ, USA). The ammonium metatungstate hydrate (H_26_N_6_O_40_W_12_·*x*H_2_O) purchased from Sigma-Aldrich™ Chemie GmbH (Steinheim, Germany) was used as a raw material to generate WO_3 _at high temperatures. Methyl orange (MO, C_14_H_14_N_3_NaO_3_S, 99.9%, Duksan Pure Chemical Co., Ltd) was of analytical grade.

### Preparation of WO_3_-fullerene composites

MCPBA (*m*-chloroperbenzoic acid, ca. 1 g) was suspended in 50 ml benzene, followed by the addition of fullerene (ca. 30 mg). The mixture was heated under reflux in air and stirred for 6 h at 343 K. The solvent was then dried at the boiling point of benzene (353.13 K). After completion, the dark brown precipitates were washed with ethyl alcohol and dried at 323 K, resulting in the formation of oxidized fullerene. For WO_3 _coating, 3.8 × 10^-5 ^mol H_26_N_6_O_40_W_12_·*x*H_2_O was added to 50 ml of distilled water (shown in Table 1). The resulting mixture was heated under reflux in air and stirred at 343 K for 6 h using a magnetic stirrer in a vial. After heat treatment at 773 K for 1 h, the WO_3_-fullerene compounds were formed.

**Table 1:** Nomenclature of the samples prepared with the photocatalysts

Preparation method	Nomenclatures
3.8 × 10^-5 ^mol H_26_N_6_O_40_W_12_·*x*H_2_O + H_2_O + MCPBA + 30 mg fullerene	WO_3_-fullerene
MCPBA+ benzene + 30 mg fullerene + 3 ml TNB	Fullerene-TiO_2_
MCPBA+ benzene + 30 mg fullerene + 3.8 × 10^-5 ^mol H_26_N_6_O_40_W_12_·*x*H_2_O + H_2_O + benzene + 3 ml TNB	WO_3_-fullerene/TiO_2_

### Preparation of WO_3_-fullerene/TiO_2 _composites

WO_3_-fullerene was prepared using pristine concentrations of TNB for the preparation of WO_3_-fullerene/TiO_2 _composites. WO_3_-fullerene powder was mixed with 3 ml TNB. The solutions were homogenized under reflux at 343 K for 5 h, while being stirred in a vial. After stirring, the solution transformed to WO_3_-fullerene/TiO_2 _gels and heat treated at 873 K to produce the WO_3_-fullerene/TiO_2 _composites.

### Characterization of photocatalysts compounds

To measure the structural variations, XRD patterns were obtained using an X-ray generator (Shimadzu XD-D1, Shimadzu Corporation, Kyoto, Japan) with Cu Kα radiation. Scanning electron microscopy (SEM, JSM-5200, JEOL, Tokyo, Japan) was used to observe the surface state and structure of the photocatalyst composites. Energy dispersive X-ray spectroscopy (EDX) was also used for elemental analysis of the samples. The specific surface area (BET) was determined by N_2 _adsorption measurements at 77 K (Monosorb, Quantachrome Instruments Ltd, Boynton Beach, FL, USA). Transmission electron microscopy (TEM, JEM-2010, JEOL) was used to observe the surface state and structure of the photocatalyst composites at an acceleration voltage of 200 kV. TEM was also used to examine the size and distribution of the titanium and iron particles deposited on the fullerene surface of various samples. The TEM specimens were prepared by placing a few drops of the sample solution on a carbon grid. UV-vis diffused reflectance spectra were obtained using a UV-vis spectrophotometer (Neosys-2000, Scinco, Seoul, South Korea) by using BaSO_4 _as a reference and were converted from reflection to absorbance by the Kubelka-Munk method.

### Photocatalytic degradation of MO

The photocatalytic activities were evaluated by MO degradation in aqueous media under visible light irradiation. For visible light irradiation, the reaction beaker was located axially and held in a visible lamp (8 W, halogen lamp, KLD-08L/P/N, Fawoo Technology, Bucheon Si, South Korea) box. The luminous efficacy of the lamp is 80 lm/W, and the wavelength is 400 nm to approximately 790 nm. The lamp was used at a distance of 100 mm from the aqueous solution in a dark box. The initial concentration of the MO was set at 1 × 10^-5 ^mol/L in all experiments. The amount of the photocatalysts (WO_3_-fullerene, fullerene-TiO_2_, and WO_3_-fullerene/TiO_2_) composite was 0.05 g per 50 ml solution. The reactor was placed for 2 h in the darkness box in order to make the photocatalyst composites particles adsorbed the MO molecule maximum. After the adsorption state, the visible light irradiation was restarted to make the degradation reaction proceed. In the process of degradation of methyl orange, a glass reactor (diameter = 4 cm, height = 6 cm) was used and the reactor was placed on the magnetic churn dasher. The suspension was then irradiated with visible light for a set irradiation time. Visible light irradiation of the reactor was done for 10, 30, 60, 90, and 120 min, respectively. Samples were withdrawn regularly from the reactor and dispersed powders were removed by a centrifuge. The clean transparent solution was analyzed by UV/vis spectroscopy. The MO concentration in the solution was determined as a function of the irradiation time.

### Elemental analysis of the preparation

**Figure 1:**
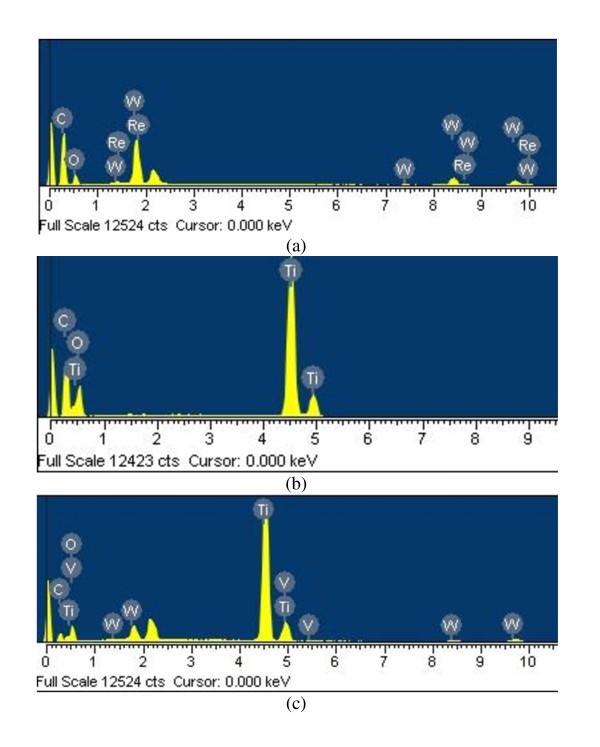
**EDX elemental microanalysis of WO_3_-fullerene, fullerene-TiO_2_, and WO_3_-fullerene/TiO_2_**.

Figure 1 shows the EDX patterns of the WO_3_-treated fullerene, fullerene-supported TiO_2_, and WO_3_-fullerene/TiO_2_. EDX indicated C, O, Ti, and W as the major elements in the composites. Table 2 lists the numerical results of EDX quantitative microanalysis of the samples. Figure 1c shows the presence of C, O, and Ti, as major elements with strong W peaks. There were some small impurities, which were attributed to the use of fullerene without purification. In most samples, carbon and titanium were present as major elements with small quantities of oxygen in the composite.

**Table 2:** EDX elemental microanalysis, BET surface area, and *k*_app _values of photocatalysts

Sample name	*C *(%)	*O *(%)	*W *(%)	Impurity (%)	Ti (%)	BET (m^2^/g)	*k*_app_
C_60_	99.99	-	-	0.01	-	85.05	-
TiO_2_	-	-	-	0.01	99.99	18.95	2.24 × 10^-4^
WO_3_-fullerene	54.08	17.25	22.92	5.75	-	73.25	2.86 × 10^-3^
Fullerene-TiO_2_	27.24	36.71	-	0.02	58.82	64.62	1.52 × 10^-3^
WO_3_-fullerene/TiO_2_	10.41	35.28	3.22	1.03	50.06	57.74	4.75 × 10^-3^

### Surface characteristics of the samples

Table 2 lists the specific surface area (BET) of the materials examined. The BET surface area of pure TiO_2 _was 18.95 m^2^/g, and the surface area of pure fullerene was 85.05 m^2^/g. Tungsten oxide particles were introduced into the pores of fullerene, which decreased the BET surface area. The surface area of fullerene-TiO_2 _was 64.62 m^2^/g. Fullerene contains many pores, which can increase the surface area of the photocatalyst. The BET surface area decreased from 85.05 m^2^/g for pure fullerene to 57.74 m^2^/g for WO_3_-fullerene/TiO_2_. This suggests that the TiO_2 _and tungsten oxide were introduced into the pores of the fullerenes, which decreased the BET surface area. The WO_3_-fullerene sample had the largest surface area, which can affect the adsorption reaction.

**Figure 2:**
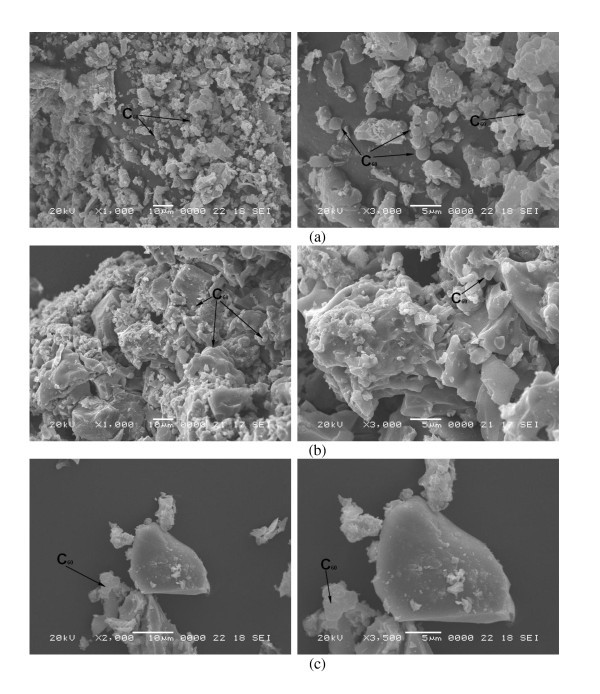
**SEM images of WO_3_-fullerene (a), fullerene-TiO_2 _(b), and WO_3_-fullerene/TiO_2 _(c)**.

The micro-surface structures and morphology of the fullerene-TiO_2_, WO_3_-fullerene, and WO_3_-fullerene/TiO_2 _composites were characterized by SEM (Figure 2). SEM is used for inspecting topographies of specimens at very high magnifications using a piece of equipment called the scanning electron microscope. Figure 2 shows the macroscopic changes in the morphology of the WO_3_-fullerene, fullerene-TiO_2_, and WO_3_-fullerene/TiO_2_. In Figure 2a, WO_3_-fullerene has the small particle size and a good dispersion. The fullerene particles were spherical particles in shape with small facets, and fullerene has a good dispersion [18]. For the fullerene-TiO_2 _sample (Figure 2b), the fullerene particles were well attached to the TiO_2 _surface with a uniform distribution, but the particle size is bigger than WO_3_-fullerene. Zhang et al. reported that a good dispersion of small particles could provide more reactive sites for the reactants than aggregated particles [19]. At the same time, the conductivity of fullerene can facilitate electron transfer between the adsorbed dye molecules and catalyst substrate. With the WO_3_-fullerene/TiO_2 _samples (Figure 2c), tungsten particles were fixed to the TiO_2 _surface and fullerene particles in some spherical particles, but the distribution was not uniform. There was no clear difference in the intensity of aggregation. Because of the aggregation, fullerene cannot show clearly. The particles were strongly aggregated and that discrete particles were impossible to find so the average particle size was difficult to obtain. It may be that particles with similar or close crystallographic orientations were formed bulky crystal or quasi-crystals with modulated surfaces and regular shapes.

**Figure 3:**
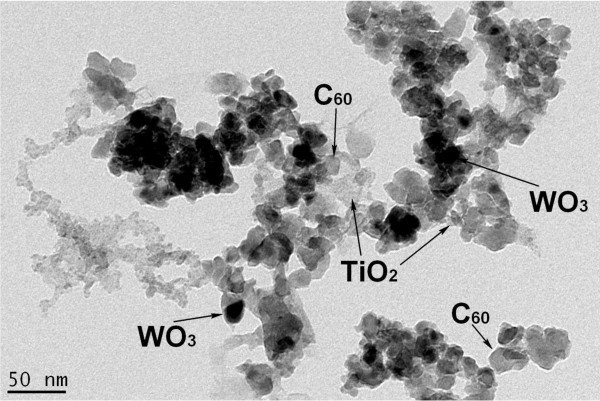
**TEM image of the WO_3_-fullerene/TiO_2 _composites**.

Figure 3 shows TEM images of the WO_3_-fullerene/TiO_2 _composites. TEM is a technique used for analyzing the morphology, crystallographic structure, and even compositing of a specimen. As shown in Figure 3, particles were observed upon enlargement of the images. This indicates that the surface of the WO_3 _particles is cleaned under exposure to the reaction conditions. Figure 3 shows large clusters with an irregular agglomerated dispersion of TiO_2_. Fullerene were distributed uniformly outside the surface of the TiO_2 _nanoparticles with a size of approximately 10 to 20 nm, and WO_3 _were distributed uniformly over the surface of the fullerene and TiO_2_, even though this caused partial agglomeration to form block particles. TEM also revealed the presence of metal nanoparticles on the fullerene particles.

### Structural analysis

**Figure 4:**
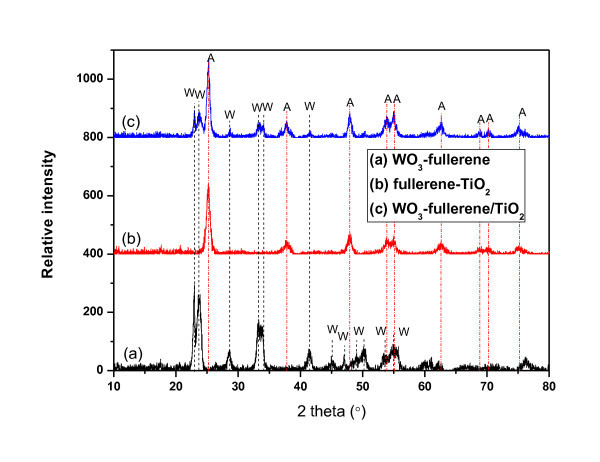
**XRD patterns of WO_3_-fullerene (a), fullerene-TiO_2 _(b), and WO_3_-fullerene/TiO_2 _(c)**.

XRD was used to determine the crystallographic structure of the inorganic component of the composite. Figure 4 shows the XRD patterns of the WO_3_-treated fullerene, fullerene-supported TiO_2_, and WO_3_-fullerene/TiO_2_. In Figure 4, A is anatase and W is the monoclinic phase of tungsten oxide. The structure of WO_3_-fullerene composites showed monoclinic phase of tungsten oxide. The peaks at 23.15°, 23.61°, 24.37°, 26.61°, 33.33°, 33.65°, 34.01°, 41.51°, 44.88°, 47.22°, 49.32°, 50.48°, 53.46°, and 55.11° 2*θ *were assigned to diffraction planes of (001), (020), (200), (120), (111), (021), (201), (220), (221), (131), (002), (400), (112), (022), and (401) of monoclinic WO_3 _phase [20,21]. WO_3_-fullerene/TiO_2 _and fullerene-TiO_2 _showed anatase phase of TiO_2_. The crystal structure of TiO_2 _is determined mainly by the heat-treated temperature. The peaks at 25.3°, 37.5°, 48.0°, 53.8°, 54.9°, and 62.5° 2*θ *were assigned to the (101), (004), (200), (105), (211), and (204) planes of anatase [22-24], indicating the developed fullerene/TiO_2 _composites existed as anatase. In the XRD patterns for WO_3_-fullerene/TiO_2_, the peaks at 23.15°, 23.61°, 24.37°, 26.61°, 33.33°, 33.65°, 34.01°, and 41.51° 2*θ *were assigned to diffraction planes of (001), (020), (200), (120), (021), (201), (220), and (221) of monoclinic WO_3 _phase. Due to the small content of tungsten oxide (shown in Table 2), the intension of the peaks are smaller than that of WO_3_-fullerene, and the other peaks cannot be found in these patterns.

### UV-vis diffuse reflectance spectroscopy

**Figure 5:**
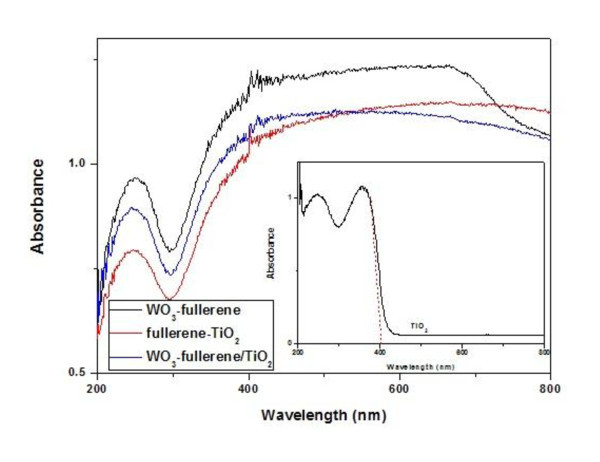
**UV-vis absorption spectra of photocatalysts**.

The UV-vis absorption spectra of the samples are shown in Figure 5; the illustration is UV-vis absorption spectra of pure TiO_2_. We can find that TiO_2_, WO_3_-fullerene, fullerene-TiO_2_, and WO_3_-fullerene/TiO_2 _composites have great absorption at ultraviolet region, but the absorption edge of TiO_2 _is approximately 400 nm (*E*_g _= 3.2 eV). When at the visible region, WO_3_-fullerene, fullerene/TiO_2_, and WO_3_-fullerene-TiO_2 _composites have good absorption; this is also means that these composites have great photocatalytic activity under visible light irradiation. Because WO_3 _has a relatively small band gap (2.6 eV to approximately 3.0 eV), WO_3 _have photocatalytic activity at visible region, from the wavelength at 400 to 443 nm. And fullerene also acted as a photosensitizer, so that WO_3_-fullerene has good adsorption at visible region. In the case of fullerene-coupled TiO_2_, fullerene acted as a photosensitizer, which could be excited to inject electrons into the conduction band of TiO_2_. Because of the synergistic reaction of WO_3_, fullerene, and TiO_2_, the adsorption effect of WO_3_-fullerene/TiO_2 _is good at visible region [25,26].

### Photocatalytic activity of samples

**Figure 6:**
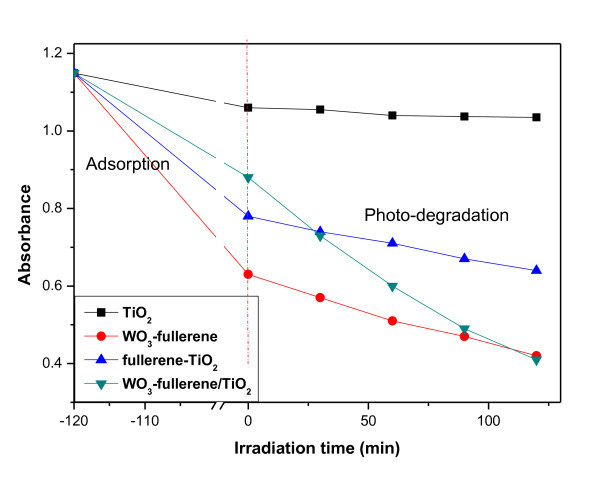
**Decolorization effect on MO of pure TiO_2_, WO_3_-fullerene, fullerene-TiO_2_, and WO_3_-fullerene/TiO_2_**.

Two steps are involved in the photocatalytic decomposition of dyes, the adsorption of dye molecules, and their degradation. After adsorption in the dark for 2 h, all the samples reached adsorption-desorption equilibrium [27]. Figure 6 shows the adsorptive and degradation effect of photocatalysts for MO. In the adsorptive step, TiO_2_, WO_3_-fullerene, fullerene-TiO_2_, and WO_3_-fullerene/TiO_2 _composites showed different adsorptive effects with WO_3_-fullerene having the best adsorptive effect, and the adsorptive effect of pure TiO_2 _was the lowest. This is because fullerene can enhance the adsorption effect. WO_3_-fullerene has the largest BET surface area, which will affect the adsorptive effect. The decolorization efficiencies of WO_3_-fullerene, fullerene-TiO_2_, and WO_3_-fullerene/TiO_2 _composites were 45.17%, 32.12%, and 23.41%, respectively. These results are consistent with the BET surface areas.

In the degradation step, Figure 6 shows the results of TiO_2_, WO_3_-fullerene, fullerene-TiO_2_, and WO_3_-fullerene/TiO_2 _composites degradation MO solutions under visible light. The relative yields of the photolysis products formed under different irradiation time conditions are shown for the products. The dye concentration was 1.0 × 10^-5 ^mol/l, and the absorbance decreased with increasing irradiation time. This suggests that the light transparency of the dye concentration was increased greatly by the photocatalytic degradation effect. The effect of the high crystallinity of the anatase phase on the photocatalytic degradation of dye was shown. Under visible light irradiation, TiO_2 _cannot depredate MO molecules, but WO_3_-fullerene, fullerene-TiO_2_, and WO_3_-fullerene/TiO_2 _composites have good photocatalytic activity. Comparing these three samples, WO_3_-fullerene/TiO_2 _composite has the best degradation effect, which is due to the synergistic reaction of WO_3_, fullerene, and TiO_2_.

**Figure 7:**
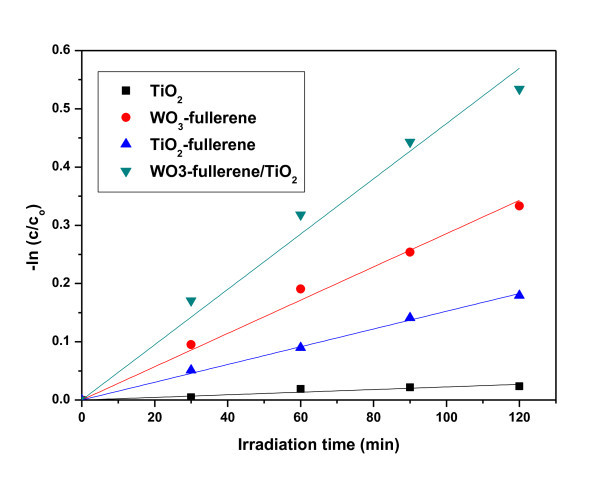
**Corresponding -ln(*C*/*C*_0_) vs. *t *plots**.

Figure 7 presents the corresponding -ln(*C*/*C*_0_) vs. *t *plots at 0 to 120 min irradiation time. The photodegradation followed first-order kinetics. The kinetics can be expressed as follows: -ln(*C*/*C*_0_) = *k*_app_*t*, where *k*_app _is the apparent reaction rate constant, and *C*_0 _and *C *are the initial concentration and the reaction concentration of MO, respectively. Table 2 shows the rate constant values (*k*_app_) of pure TiO_2_, WO_3_-fullerene, fullerene-TiO_2_, and WO_3_-fullerene/TiO_2 _composites for the degradation of the MO solution. The *k*_app _value of the WO_3_-fullerene/TiO_2 _sample is the largest, which is in accord with the photocatalytic activity.

**Figure 8:**
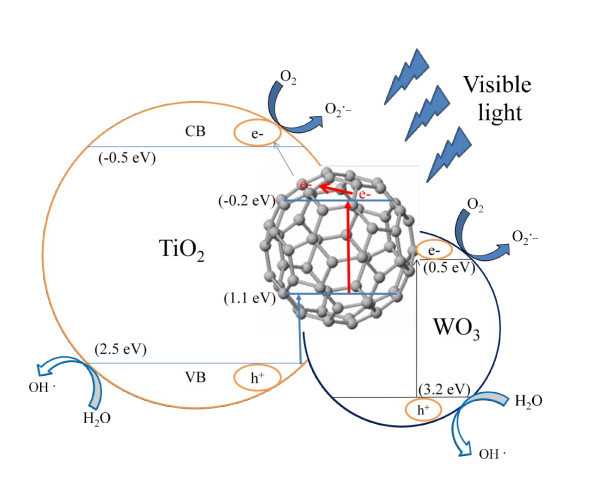
**Schematic diagram of the separation of photogenerated electrons and holes on the WO_3_-fullerene/TiO_2 _interface**.

Fullerene-TiO_2 _has a better degradation effect than pure TiO_2 _because fullerene is an energy sensitizer that improves the quantum efficiency and increases charge transfer [28,29]. The TiO_2 _deposited on the fullerene surface can retain its photodegradation activity. In the fullerene-coupled TiO_2 _system, the photocatalytic activities were enhanced mainly due to the high efficiency of charge separation induced by the synergistic effect of fullerene and TiO_2_. In the case of fullerene-coupled TiO_2_, hole and electron pairs were generated and separated on the interface of fullerene by visible light irradiation. The level of the conduction band in TiO_2 _was lower than the reduction potential of fullerene. Therefore, the photogenerated electron can transfer easily from the conduction band of fullerene to a TiO_2 _molecule with an interaction between fullerene and TiO_2_. Simultaneously, the holes in the valence band (VB) of TiO_2 _can transfer directly to fullerene because the VB of TiO_2 _matches well with fullerene. The synergistic effect fullerene and TiO_2 _both promoted the separation efficiency of the photogenerated electron-hole pairs, resulting in the high photocatalytic activity of fullerene-hybridized TiO_2 _samples. In this case, the fullerene-coupled TiO_2 _system improved the reaction state [30-32]. Therefore, the fullerene-coupled TiO_2 _has photocatalytic activity under visible light. Figure 8 shows a schematic diagram of the separation of photogenerated electrons and holes on the fullerene-TiO_2 _interface.

WO_3_-fullerene also has a barrier degradation effect than pure TiO_2_, due to the same reason as fullerene-TiO_2 _system. From Figure 6 and Table 2, we can find that the *k*_app _of WO_3_-fullerene is 2.86 × 10^-3^, which is larger than that of fullerene-TiO_2 _(1.52 × 10^-3^). This is because, with the band gap of WO_3 _being relatively small, electrons will obtain energy to jump onto the conduction band and become free electrons named photoelectrons when under visible light irradiation. In this system hole and electron pairs were also generated and separated on the interface of fullerene. Fullerene is acted as photosensitize. These electron-hole pairs can recombine or diffuse to the surface where they can initiate redox reactions with surface species, so the degradation effects of TiO_2_-fullerene and WO_3_-fullerene/TiO_2 _were limited.

At WO_3_-fullerene/TiO_2 _system, the photocatalytic activities were enhanced mainly due to the high efficiency of charge separation induced by the synergistic effect of fullerene, WO_3_, and TiO_2_. Because of the least band gap of fullerene (1.6 to 1.9 eV), hole and electron pairs were generated and separated on the interface of fullerene easily by visible light irradiation, and the electron can transfer easily from the CB of fullerene to a TiO_2 _molecule and, simultaneously, the holes in the VB of TiO_2 _can transfer directly to fullerene because both the conduction band (CB) and the valence band (VB) of WO_3 _were higher than the CB and VB of TiO_2 _and fullerene. When the hole and electron pairs were also generated and separated on the interface of WO_3_, electrons at the CB of WO_3 _migrated to CB of TiO_2 _and fullerene, and holes at the VB of WO_3 _migrated to VB of TiO_2 _and fullerene [33]. This can allow the transfer of photogenerated electrons facilitating effective charge separation and decreased the rate of recombination about the electron-hole pairs. Fullerene also acts as the adsorb facient and increases the surface area of compounds which can increase the adsorption effect for samples, adsorbed more O_2 _and dye molecules, and make sure this systems take full advantage of yield oxidizing species. Figure 8 is the schematic diagram of the separation of photogenerated electrons and holes on the WO_3_-fullerene/TiO_2 _interface. Electrons and holes were used to produce the hydroxyl radicals (OH^·^) and superoxide ions (O_2_^·-^). Oxidative degradation of azo dyes occurs by the attack of hydroxyl radicals and superoxide ions, which are the highly reactive electrophilic oxidants. Due to the efficiency of hydroxyl radicals and superoxide ions, azo dyes were decompounded to CO_2_, H_2_O, and inorganic.

## Conclusions

This study examined the preparation and characterization of WO_3_-fullerene, fullerene-TiO_2_, and WO_3_-fullerene/TiO_2_. The BET surface area of pristine fullerene was higher than that of the WO_3_-fullerene/TiO_2 _composite. XRD revealed the WO_3 _structure and anatase. TEM showed that TiO_2 _particles with some agglomerates were dispersed over the surface of fullerene together with WO_3 _particles. In UV-vis absorption, spectra samples have shown a great adsorption at visible region. Fullerene-TiO_2 _has a good photodegradation effect under visible light irradiation, due to the photosensitivity, and enhances the BET surface area effect of fullerene. The WO_3_-fullerene/TiO_2 _composite showed the best photocatalytic degradation activity of the MO solution under visible light irradiation. This was attributed to the three different effects between the photocatalytic reactions of the supported TiO_2_, to the energy transfer effects of fullerene and WO_3_, such as electrons and light, and to the separation effect in this system.

## Competing interests

The authors declare that they have no competing interests.

## Authors' contributions

The work presented here was carried out in collaboration between all authors. WCO an MZD defined the research theme. MZD and WCO designed methods and experiments, and experiments and wrote the paper. LZ carried out the laboratory experiments. JGC and CYP analyzed the date, interpreted the results. All authors have contributed to, seen and approved the manuscript.
